# Latin Prescriptions

**Published:** 1846-06-01

**Authors:** 


*Latin Prescriptions.*—We have noticed, of late, several articles in foreign and domestic journals, respecting the absurdity of perpetuating the pedantic habit of writing prescriptions in Latin. We hope the result will be, that it will be considered antiquated nonsense to use aught beside our own vernacular, in the directions for the apothecary or patient. The sooner the profession is stripped of all remnants of affectation and mystery, the better. For the sake of uniformity, however, and to avoid confusion, it will be advisable to continue the present system of forming the nomenclature of the pharmacopœia and materia medica, from Latin or Greek derivatives. The objection is to tailing the formula with mongrel Latin, when pure English would answer quite as well, and certainly be in better taste. We believe the majority of the physicians of this country, have already discarded the practice. We occasionally, however, see it persisted in published reports, when, in many cases, it is to be presumed, it has cost the writer considerable pains to appear ( as he doubtless thinks ) quite scholastic.

